# Changes in physical performance, body composition and physical training during military operations: systematic review and meta-analysis

**DOI:** 10.1038/s41598-023-48712-2

**Published:** 2023-12-05

**Authors:** K. Pihlainen, M. Santtila, B. C. Nindl, J. Raitanen, T. Ojanen, J. P. Vaara, J. Helén, T. Nykänen, H. Kyröläinen

**Affiliations:** 1 Human Performance Sector, Training Division, Defence Command, Helsinki, Finland; 2 Department of Leadership and Military Pedagogy, National Defence University, Helsinki, Finland; 3 Neuromuscular Research Laboratory/Warrior Human Performance Research Center, Department of Sports Medicine, School of Health and Rehabilitation Sciences, University of Pittsburgh, Pittsburgh, PA USA; 4 Faculty of Social Sciences (Health Sciences), Tampere University, Tampere, Finland; 5 UKK Institute for Health Promotion Research, Tampere, Finland; 6 Human Performance Division, Finnish Defence Research Agency, Tuusula, Finland; 7 Army Academy, Finnish Defence Forces, Lappeenranta, Finland; 8 Neuromuscular Research Center, Faculty of Sport and Health Sciences, University of Jyväskylä, Jyvaskyla, Finland

**Keywords:** Physiology, Musculoskeletal system

## Abstract

Systematic review and meta-analysis applying PRISMA guidelines with a PICOS format was constructed to provide an overview of changes in physical performance, body composition and physical training in soldiers during prolonged (≥ 3 months) military operations. Twenty-four studies out of the screened 4431 records filled the inclusion criteria. A small decrease in endurance performance was the most consistent finding (Hedge's g [*g*] − 0.21, 95% CI − 0.01 to − 0.41) while small overall increases in maximal strength of the lower (*g* 0.33, 95% CI 0.16–0.50) and upper body (*g* 0.33, 95% CI 0.19–0.46) were observed. In addition, small increases in strength endurance (push-up, g 0.34, 95% CI 0.15–0.52; sit-up g 0.26, 95% CI 0.07–0.44) were observed. The overall changes in body composition were trivial. Heterogeneity in the outcome variables varied mainly between low to moderate. Large inter-individual variations were observed in physical training volume, including decrements especially in endurance training frequency and volume. A reduction in total training load was often associated with negative changes in body composition and physical performance according to the principle of training specificity. Individuals with higher initial fitness level were more susceptible to decrements in their physical performance during operation.

## Introduction

Arduous occupational physical demands in various military environments are widely acknowledged. Ground force soldiers commonly perform their occupational duties wearing combat load in a prolonged manner at low intensities which increases energy expenditure mainly through aerobic metabolism. However, duties may also include intensive phases (e.g., combat actions, casualty evacuation, repetitive lifting) which raise the physical activity unexpectedly to very high levels^1–4^, requiring higher neuromuscular performance and anaerobic energy production^5,6^. Under such conditions, soldiers may not have sufficient time for recovery and could thus experience accumulation of physiological stress leading to fatigue. Acute physical fatigue deteriorate cognitive function, physical performance and critical combat skills such as shooting accuracy^7–11^. In general, dramatic short-term hormonal disturbances and negative changes in body composition as well as physical performance have been reported following demanding military field training^3,12–15^.

Occupational physical demands typically increase during military operations. According to Nagai et al.^16^, 58% of combat aviation support soldiers carried regularly external loads with an average load of 22 kg during a one-year deployment in Afghanistan. The average duration and frequency of load carriage tasks were 3.5 h and 3.7 days per week. Boye et al.^17^ reported that the prevalence of soldiers performing physically demanding tasks during deployment increased from an average of 12 to 22% when compared to garrison duties. On the other hand, physical training decreased during deployment by 6% (17% vs. 11%), respectively. The duration for an individual soldier being deployed may easily span over several months^16,18–21^ and during that time, physical performance may deteriorate significantly with detraining superimposed under military operational stress. For example, decreases of up to 15–20% in maximal aerobic fitness (VO_2_max)^22^ and about 10% in maximal muscular strength^23^ have been observed already four weeks after the cessation of training. Detraining is an important consideration for physically demanding occupations, since a decline in an individual’s physical fitness increases the relative physiological demands of performing a task, reduces overall working capacity during prolonged assignments, and thereby increases the risk of injury^24^.

High level of physical fitness and optimal body composition in combination with required occupational skills are significant factors for success in an military operational and deployed environment. The importance of endurance performance increases with the duration of the physically demanding task, such as load carriage^4,25^. Higher endurance performance has also been associated with better stress tolerance and improved ability to maintain cognitive performance^8,11,25,26^ While endurance performance is associated with military tasks of longer duration, stronger relationships have been observed between high load, short duration tasks and muscular strength and power^4,27^. In addition, a lower amount of fat mass and higher muscle mass have been reported to be associated with improved occupational performance for various military tasks^28–30^. In addition to having higher occupational performance capacity, physically fit soldiers may be more resilient to operational stressors in demanding military environments^31^. This is partly explained by improved sensitivity of the neuroendocrine system leading to the ability to recover faster from high operational stress^32^.

Several studies have shown beneficial effects of physical training on military task performance^4^. From a career perspective, military performance optimization starts from initial entry recruit or basic training. The following employment training aim to build up a basic level of strength and endurance capacity for recruits, so that minimum standards for deployment are met^33^. Before the deployment, fitness level can be further enhanced so that peak performance may be reached by the time the soldiers are transported to the operational area^33,34^. The deployment phase can be compared to the competitive period of an athlete, with the aim of maintenance of physical performance qualities, which have been achieved during preparation period, throughout the operation^33,34^. However, optimal methods of developing or maintaining physical and occupational performance during a prolonged military deployment are still under debate. While many studies have evaluated training adaptations in non-deployed soldiers, limited information is available from prolonged military deployments^3^. Therefore, the aim of the present review and meta-analysis is to provide an overview of physical training and changes in body composition, physical performance, as well as their relationships, during prolonged military operations. More specifically, the aim is to review changes in physical performance, body composition and physical training that have been observed in soldiers during longer (≥ 3 months) military deployments and how physical training during deployment impacts changes in physical performance and body composition outcomes. The rationale for this review is to provide knowledge and suggestions for subject matter experts on how physical training should be taken into consideration during prolonged military operations in order to avoid the deleterious effects of detraining and decrements in combat readiness.

## Methods

This review and meta-analysis was constructed applying the PRISMA guidelines^35^ for methodology and reporting. The participant, intervention, comparator, outcome, study design (PICOS) format^36^ was used to develop eligibility criteria for study inclusion (Table 1). English-written peer-reviewed journal articles with a minimum of 3-month pre-post follow-up design for body composition and physical performance variables in deployed military personnel were eligible to the review. In addition, all available during-deployment training related data were taken into consideration.

**Table 1:** Participant, intervention, comparator, outcome, study design (PICOS) inclusion and exclusion criteria.

	Inclusion criteria	Exclusion criteria
Population	Healthy deployed land-operating (Army) military personnel	Non-deployed military personnel
Intervention	Deployment with a minimum duration of 3 months	Deployments shorter than 3 months
Comparator	NA	NA
Outcomes	Objectively measured body composition and/or physical performance variables	Self-reported physical fitness or body composition data Studies with only self-reported training data
Study design	Empirical longitudinal (pre-post) studies Peer-reviewed journal article or review	Cross-sectional studies Book, technical report, congress proceeding, etc

*NA* not applicable.

Data for the present review were collected during November 2023 using Medline (PubMed), Google Scholar, and Scopus databases in the mentioned order. The boolean search query ““change*” AND (“body composition” OR “fitness” OR “exercise”) AND (“deployment” OR “military operation”)” was used for screening. In addition, citation search from the records filling the inclusion criteria was performed. Flowchart of the search strategy is presented in Supplement Table S1.

The screening of articles for potential relevance was firstly determined based on the title of the article, and secondly on abstract. The abstract screening and the inclusion selection was performed by two independent researchers who also were subject matter experts in the field of soldier physical performance in different military environments. Possible contradictory opinions on inclusion were decided by a third researcher who carefully read the articles and made the final decision for inclusion or exclusion. Finally, the abstract-screened full text articles were obtained and read, and the relevant ones were included in the review. Additionally, the references from the full text articles were reviewed for potential additional papers. After full-text screening phase, participant demographics, methodological design and main findings regarding physical activity and training, as well as changes in physical performance and body composition were compiled into Table 2.

**Table 2:** Main findings of the reviewed studies.

Study	N (descriptives), operation, duration, delay between deployment and post measurement	Physical activity/training	Changes (pre-post) in body composition	Changes (pre-post) in physical performance	Relationships/associations
Dyrstad et al.^18^	n = 71 males (21 ± 2 yrs, 80.3 ± 9.7 kg, 181 ± 7 cm), Kosovo peacekeeping operation, 12 months, measurement delay 0 days	Training frequency and volume 1.8 ± 1.2 sessions and 117 ± 77 min/week, respectively (endurance training 0.6 ± 0.5 sessions/32 ± 31 min; strength training 1.3 ± 1.0 sessions/85 ± 70 min)	Body mass + 3%	3 km run time + 5% RM sit-ups, NS RM push-ups, NS RM pull-ups + 38%	Operation training volume versus ∆ VO_2_max, r = 0.46, *p* < 0.001
Sharp et al.^19^	n = 110 (23 ± 5 yrs, 83.3 ± 14.7 kg, 178 ± 7 cm), Afghanistan, 9 months, measurement delay 18 ± 14 days (range 5–209 days)	Percent of soldiers training ≥ 3 times/week (pre vs during): Endurance: 80% versus 35% Strength: 58% versus 56% Sports: 11% versus 8% Percent of soldiers training > 30 min/week (pre vs during): Endurance: 78% versus 57% Strength: 90% versus 74% Sports: 54% versus 53%	Body mass − 2% Fat-free mass − 4% Body fat + 8% Body fat + 10% Bone mineral content − 4%	VO_2_max -5% 2 kg medicine ball throw − 5% Vertical jump, NS Dynamic lifting strength, NS	Pre versus during ∆ strength training frequency versus ∆ fat-free mass, r = 0.37, *p* < 0.01,
Lester et al.^54^	n = 73 males (24 ± 5 yrs, 76.6 ± 10.2 kg, 174 ± 7 cm), Iraq, 13 months, measurement delay max. 14 days	Percent of soldiers training ≥ 3 times/week (pre vs during): Endurance: 88% versus 29% Strength: 63% versus 44% Sports: 85% versus 34% Percent of soldiers training > 30 min/week (pre vs during): Endurance: 85% versus 41% Strength: 81% versus 69% Sports: 85% versus 34%	Body mass + 3% Lean mass + 3% Body fat + 9% Body fat + 4%	2 mile run time + 13% 1RM bench press + 7% 1RM squat + 8% 30%1RM bench throw + 9% Squat jump, NS	Operation strength training frequency versus ∆ 1RM bench press, r = 0.61, *p* < 0.05, Operation strength training duration versus ∆ 1RM bench press, r = 0.48, *p* < 0.05, Operation strength training frequency versus ∆ lean mass, r = 0.34, *p* < 0.001
Warr et al.^51^	n = 54 (47 males and 7 females, 27 ± 7 yrs, 82.9 ± 15.8 kg, 174 ± 7 cm), Afghanistan, Iraq, 10–15 months, measurement delay max. 10 days	NA	Body mass − 2% Body fat − 11%	VO_2_max − 11% 1RM bench press relative to body mass + 10% 1RM squat relative to body mass + 14% 2 min push-up + 16% 2 min sit-up + 11% Wingate anaerobic power, NS	Operation medical visits versus ∆ VO_2_max; back-related visits, r = − 0.31, *p* < 0.05, behavioral health visits, r = − 0.28, *p* < 0.05
Rintamäki et al.^55^	n = 20 males (22 ± 3 yrs, 78.8 ± 11.5 kg, 180 ± 6 cm), Chad crisis management operation, 4 months, measurement delay NA	Percent of soldiers training ≥ 3 times/week during operation: Endurance: 11% Strength: 13%	Body mass − 4% Body fat, NS Body fat, NS	12 min running test, NS MVC_lower_ + 8% Grip strength, NS 1 min push-up, NS 1 min sit-up + 11% 1 min squats, NS Counter movement jump + 27%	NA
Carlson et al.^52^	n = 53 Combat Service Support Soldiers, 12 males, 7 females (23 ± 3 yrs, 73.3 ± 12.3 kg, 173 ± 11 cm), Iraq, 12 months, measurement delay max. 60 days	Baecke Habitual Physical Activity Index pre versusduring: Work: 2.51 versus 2.24 (− 11%) Sport: 2.53 versus 2.55 (NS) Leisure: 2.51 versus 2.58 (NS)	Body mass, NS Bone mineral density; Femoral neck + 1% Spine, NS	NA	NA
Warr et al.^53^	n = 88, 76 males (27 ± 6 yrs, 86.9 ± 14.9 kg, 177 ± 6 cm) and 12 females (32 ± 12 yrs, 66.2 ± 10.1 kg, 164 ± 5 cm), Afghanistan, Iraq, measurement delay max. 10 days	Percent of soldiers training ≥ 3 times/week during operation: Endurance: 57% (males 53%, females 83%) Strength: 67% (males 68%, females 58%)	Body mass − 2% Fat-free mass + 2% Body fat − 18% Body fat − 16%	VO_2_max, NS 1RM bench press relative to body mass + 11% 1RM squat relative to body mass + 14%	NA
Fallowfield et al.^20^	n = 249 (28 ± 7 yrs, 82.8 ± 9.1 kg, 179 ± 6 cm), Afghanistan, 6 months, measurement delay max. 7 days	NA	Body mass, NS Fat-free mass, NS Body fat, NS	VO_2_max, NS 2 min press-up, NS 2 min sit-up, NS Grip strength, NS Isometric lifting strength, NS	NA
Psutka et al.^48^	n = 251 (183 males, 68 females, 32–34 yrs, 80.3 ± 13.5 kg), Afghanistan, 3–4 months, measurement delay 0 days	NA	Body mass, NS Body fat, NS (Visceral fat − 2%) Muscle mass, NS	NA	NA
Frank et al.^56^ (Within-group significances not reported)	n = 158; Telehealth group (TG, n = 66, 24 yrs, 82.1 ± 14.7 kg, 176 ± 7 cm), Control group (CG, n = 92, 24 yrs, 85.3 ± 13.8 kg, 176 ± 7 cm), Afghanistan 9 months, measurement delay max. 30 days	Baecke Habitual Physical Activity Index pre versusduring: Work, TG: 3.35 versus 3.32 Work, CG: 3.14 versus 3.14 Sport, TG: 2.68 versus 2.97 Sport, CG: 2.94 versus 2.76* Leisure, TG: 3.14 versus 3.37 Leisure, CG: 3.28 versus 3.24 *, between-group comparison (*p* < 0.05)	TG vs CG: Body mass, NS Waist circumference, NS Body fat% + 23% versus − 2%* Bone mineral density, NS *, between-group comparison (*p* < 0.01)	NA	NA
Nagai et al.^16^	n = 35 (30 males, 5 females, 25 ± 5 yrs, 76 ± 14 kg, 174 ± 9 cm) Afghanistan, 11–12 months, measurement delay 23 ± 22 days	85% of soldiers reported engaging in physical activity regularly (endurance type of activity 82%, strength 86%, both types 71%, sports 7%) Physical training/activity frequency 4.6 ± 1.5 days/week; duration 66.8 ± 26.2 min	Body mass, NS Body fat, NS	VO_2_max, NS Anaerobic power + 7% Anaerobic capacity, NS Knee extension, NS Knee flexion, NS	NA
Farina et al.^57^	n = 50 males (34 ± 5 yrs, 86 ± 9 kg), Afghanistan or N/A, 3–6 months, measurement delay max. 10 days	Endurance training volume (pre vs during): 156 vs 250 min/week; Strength training volume (pre vs during): 190 vs 336 min/week;	Body mass, NS Lean mass + 1% Body fat, NS WC, NS NC, NS	Grip strength + 6%	∆ body mass versus ∆ SHBG, r = − 0.33, *p* < 0.05
Pihlainen et al.^49^	n = 79 males (30 ± 8 yrs, 79 ± 8 kg, 179 ± 7 cm), Lebanon, 6 months, measurement delay 0 days	pre-post during operation: Daily step count − 10% (9472 versus 8321 steps) Inactivity increased (MET < 1.5, 76% vs. 78%) of time awake No changes in light PA (MET 1.5–3.0, 12%) Moderate PA (MET 3–6) decreased (10% vs 9%) No changes in vigorous PA (MET > 6, 1%)	Body mass + 1% Muscle mass + 1% Fat mass, NS	NA	Inactivity (%) versus ∆ body mass, r = − 0.28, *p* < 0.05; Light PA (%) versus ∆ body mass, r = 0.26, *p* < 0.05; Moderate PA (%) versus ∆ body mass, r = 0.27, *p* < 0.05
Pihlainen et al.^50^	n = 66 males (30 ± 9 yrs, 79.4 ± 8.2 kg, 180 ± 7 cm), Lebanon crisis management operation, 6 months, measurement delay 0 days	Soldiers provided with a 2 × week combined strength and endurance training program were split into two groups based on ∆ in 3000-m running test time during operation: Improved endurance performance = HiR Decreased endurance performance = LoR Training frequency (pre vs during): HiR, endurance training 2.3 ± 1.4 versus 2.4 ± 1.0 (+ 28 ± 57%) sessions/week; LoR, endurance training 2.6 ± 1.6 versus 1.4 ± 1.1 (− 40 ± 64%) sessions/week; HiR, strength training 1.8 ± 1.4 versus 1.9 ± 1.1 (+ 9 ± 62%) sessions/week LoR, strength training 2.9 ± 1.2 versus 2.7 ± 1.5 (+ 15 ± 101%) sessions/week	Body mass HiR versus LoR, − 1.0% vs + 2.3% (*p* < 0.001*) Muscle mass HiR versus LoR, 0.5% versus 1.4% (NS) Fat mass HiR versus LoR, − 7.6% versus + 14.2% (*p* < 0.001*) *, between-group comparison method	MST time HiR versus LoR, − 14% versus − 8% (*p* < 0.01*) MVC_lower_ HiR versus LoR, + 17% versus + 8% (NS*) MVC_upper_ HiR versus LoR, + 2% versus + 2% (NS*) Standing long jump HiR versus LoR, + 1% versus − 1% (NS*) 1 min Sit-up HiR versus LoR, + 6% versus + 6% (NS*) 1 min Push-up HiR versus LoR, + 28% versus + 12% (*p* < 0.01*) RM Pull-up HiR versus LoR, + 40% versus + 43% (NS*)	Pre versus during ∆% endurance training frequency versus ∆% 3000 m running test time, r = − 0.57, *p* < 0.001 Pre versus during ∆% strength training frequency versus ∆% body mass, r = 0.42, *p* < 0.01; ∆% muscle mass r = 0.31, *p* < 0.05; ∆% fat mass, r = 0.35, *p* < 0.05 ∆% in strength-to-endurance training ratio versus ∆% body mass, r = 0.43, *p* < 0.05 ∆% 3000 m running test time versus body mass, r = 0.41, *p* < 0.01; fat mass, r = 0.53, *p* < 0.001; MST time, r = 0.48, *p* < 0.001
Sedliak et al.^21^	n = 25 males (30 ± 4 yrs, 87.8 ± 14.6 kg, 179 ± 6 cm), Afghanistan, 6 months, measurement delay max. 14 days	Physical training at least 1h x day; Endurance training 3 sessions/week; Strength training 3 sessions/week; Ball games 2 sessions/week	Body mass, NS Body fat − 10%	5 km run time in combat load − 6% 4 × 10 m run time − 3% 10 × 10 m run time, NS RM Pull-up + 60% Bench press + 9% Maximal power output during bench press, NS	Frequencies of endurance training/ball games (but not strength training) correlated with ∆ in physical performance and body composition variables (r = 0.42–0.74) ∆ 5 km run time in combat load versus ∆ body mass, r = 0.44, *p* < 0.01; ∆ fat mass, r = 0.49, *p* < 0.01 ∆ pull-up versus ∆ body mass, r = 0.60, *p* < 0.01; ∆ fat mass, r = 0.57, *p* < 0.01
Pihlainen et al.^47^	n = 78 males (29 ± 8 yrs, 79 ± 8 kg, 180 ± 7 cm), Lebanon crisis management operation, 6 months, measurement delay 0 days	Overall training frequency 3.2 ± 1.5 sessions/week (endurance training 1.7 ± 1.2 sessions/week; strength training 1.5 ± 0.9 sessions/week) Soldiers provided with a 2 × week combined strength and endurance training program = EXP, control group = CON	Body mass, NS (both groups) Muscle mass + 1% (EXP) Fat mass, NS (both groups)	3000 m run time, NS (both groups) MST time − 13% (EXP), − 13% (CON) MVC_lower_ + 13% (EXP) MVC_upper_, NS (both groups) Standing long jump − 2% (CON) 1 min Sit-up + 6% (EXP) 1 min Push-up + 15% (EXP), + 15% (CON) RM Pull-up + 30% (EXP), + 31% (CON)	Relative increases in strength training frequency, TES/SHBG ratio and decrease in MST time explained 32% of the variance in relative increase in muscle mass during operation Higher relative lower body strength training volume load and increased 3000m running test time explained 51% of the variance in relative increase in fat mass during operation Relative increases in BMI, MST time, decrease in RM pull-up and faster baseline 3000 m running test time explained 68% of the variance in relative increase in 3000 m running test time

*RM* repetition maximum; *NS* not significant; *NA* not available; *MVC*_*lower*_ maximal voluntary contraction force of the lower body; *DXA* dual-energy x-ray absorptiometry; *TG* Telehealth group; *CG* control group; *WC* waist circumference; *NC* neck circumference, *SHBG* sex-hormone binding globulin; *PA* physical activity; *MET* metabolic equivalent; *MST* military simulation test; *EXP* experimental group; *CON* control group; *MVC*_*upper*_ maximal voluntary contraction force of the upper body; *TES* testosterone; *BMI* body mass index.

The participant number, means and standard deviations of the selected outcome measures were entered into a spreadsheet for statistical analysis and Stata version 17.0 (StataCorp, College Station, Texas, USA) was utilized to conduct a meta-analysis along with the systematic review. Due to the low number of deployment studies in general, all studies filling the inclusion criteria were accepted for the review without methodological quality assessment. However, for meta-analysis, studies with overlapping datasets (e.g., same study population and variable in different articles) were eliminated, using only the data with the highest study participant number per case to avoid bias in results. A random-effects restricted maximum likelihood (REML) approach was used to assess inter-study heterogeneity via forest plots, which was formulated by pooling the data from the included studies. Standardized mean differences (i.e., effect size, ES) were calculated using the Hedges g^37^ and 95% confidence intervals (CI) according to Nakagawa et al.^38^ to determine the magnitude of pre versus post deployment differences with values of 0.2, 0.5, and 0.8 classified as small, medium, and large levels, respectively. Heterogeneity between the study samples included in the statistical analyses was assessed with values of 25%, 50%, and 75% classified as low, moderate, and high levels, respectively^39^.

The most commonly reported outcome variables were included in the meta-analysis when reported by four or more studies^40^. Physical performance variables included endurance performance (spiroergometry, running tests), maximal strength of the lower body (dynamic one repetition maximum i.e., 1RM lifting or squat, static lifting or leg press, knee extension) and the upper body (dynamic 1RM or static bench press, grip strength), lower body power production (vertical or horizontal jump, watt maximum in the Wingate test) and muscle endurance (repeated push-ups and sit-ups). The available anthropometric/body composition variables included body mass, muscle mass (e.g., fat-free mass, lean mass) and body fat (e.g., fat mass, fat percentage). The field running test results were converted into running speed e.g., meters per second for meta-analysis to enable their comparison with the other studies in one forest plot. Similarly, lower and upper body strength measures were converted from relative values (e.g., kg/body mass) to absolute (kg) using the study population mean body mass for the calculation to enable comparison between studies in one forest plot. However, measurement methods that differed significantly from each other (e.g., dynamic vs. isometric force assessment) were analyzed as sub-groups in the meta-analysis. The differences in the abovementioned measurement methods may have influence on the degree of changes and thus, ESs. In addition, the duration of deployments varied, as well as the delay between the deployment and the post-measurement (Table 2), both of which may increase bias to the results.

## Results

A total of 4431 records were retrieved from the database searches. The title-screening reduced the number of potential records to 136. Based on inclusion and exclusion criteria, 23 records were selected for the review process. In addition, a citation search from the selected records resulted in inclusion of one more study for the review. Thus, after the final screening, 22 articles and two reviews were included in the present review. However, six of the abovementioned journal articles^41–46^ were excluded as they reported overlapping data (e.g., same study population and variables) with the other studies included in the present review with larger participant number. Therefore, 16 journal articles and two reviews were included in the present review. The main findings of the abovementioned 16 articles are presented in Table 2.

The duration of the deployments varied from 3 to 15 months. Apart from three studies^20,30,47^ in which body composition and/or physical performance measurements were performed in the middle of the operation, only pre and post measurement results were reported. The post measurements were mainly conducted in homeland after return from the operation with a mean and standard deviation delay of 15.1 ± 15.5 days (median 10 days). The post-measurements were conducted during deployment in five studies^18,30,47–49^.

The number of participants (N = 1426, mean age 26.6 years, mean body mass 81.4 kg, mean height 178 cm) with the pre-post results varied from 20 to 251 between the studies published between the years of 2007 and 2022. The number of original participants reduced significantly in most studies due to voluntary withdrawal, injuries, increased occupational duties and even death in combat. The study participants were mainly male soldiers. Three studies^16,51,52^ reported that the participants included men and women but due to a low number of female participants, combined results were presented. Another two studies^19,20^ did not specify the sex of the participants and only one study^53^ reported combined results but also, men and women separately. The two latter subgroups were used independently in the ES calculations of this review. In addition, one study included a training intervention with a deployed control group^47^ and thus, these two groups were used separately for the ES calculations. Due to overlap in body composition and physical performance results with three other studies of Pihlainen et al.^46,49,50^, only the abovementioned^47^ was used for ES calculations.

**Figure 1:**
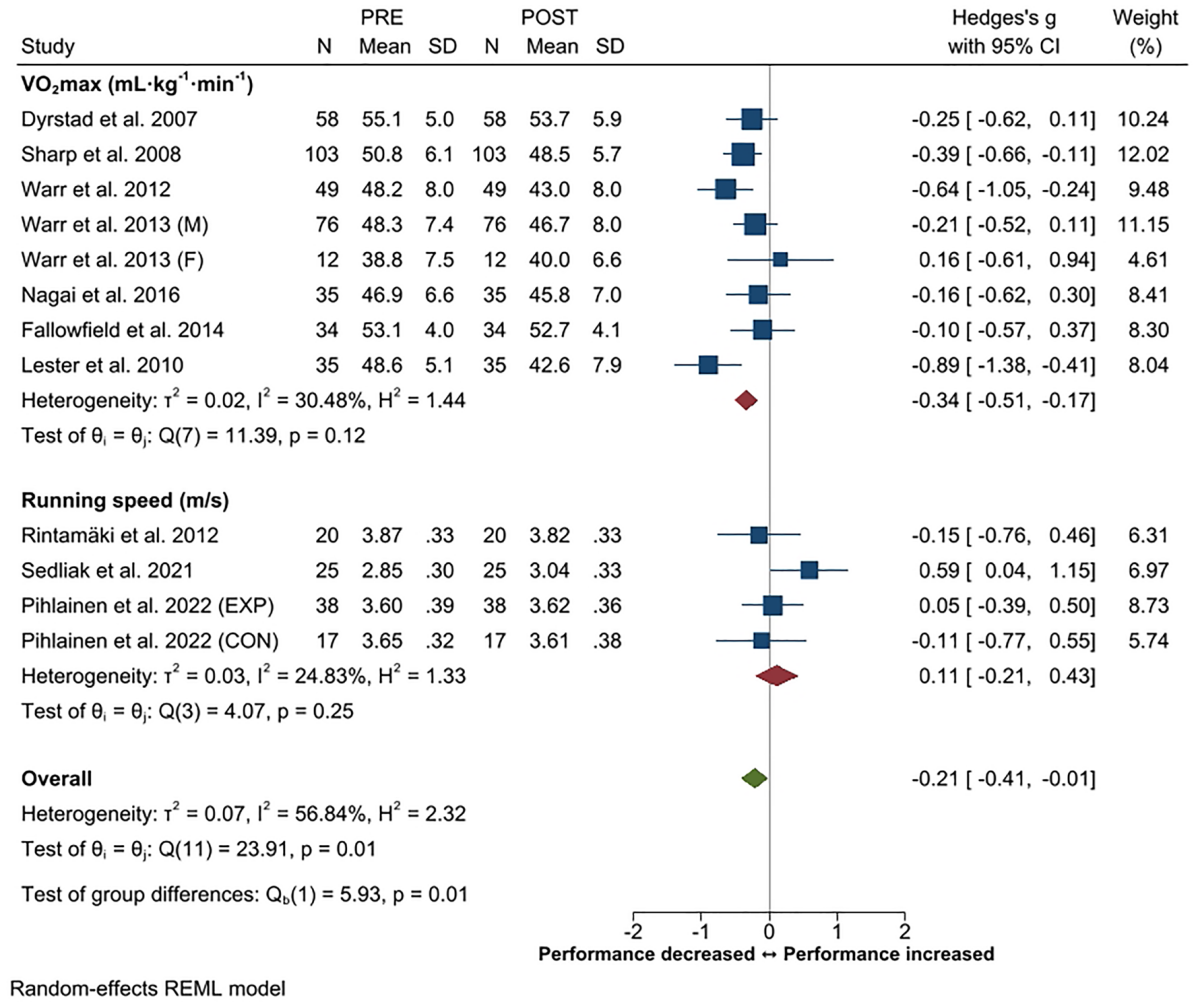
Summary of meta-analysis results for changes in endurance performance reported in standardized means (with 95% CI) and Hedge’s g. Abbreviations; M, male; F, female; EXP, experimental group; CON, control group.

A decrease in endurance performance with small but significant standardized mean difference (*g* − 0.21, 95% CI − 0.01 to − 0.41) has been the most consistently observed negative change in deployed soldiers (Fig. 1). The overall heterogeneity was moderate (57%), while in the sub-group analyses it was small. For example, Lester et al.^54^ observed a 13% decrement in the 2-mile running test performance (*g* − 0.89, 95% CI − 0.41 to − 1.38) in male soldiers deployed to Iraq for 13 months. Similarly, 10- to 15-month deployment to Iraq/Afghanistan induced an average decrease of 11% in endurance performance (*g* − 0.64, 95% CI − 0.24 to − 1.05) in 49 US Army National Guard soldiers^51^. Five studies reported no changes in endurance performance. In contrast to the abovementioned studies, Sedliak et al.^21^ reported a mean improvement of 6% (*g* 0.59, 95% CI 0.04–1.15) in 5 km run combat load run time during a six-month deployment in Afghanistan. In addition, a decrease in military specific endurance test time correlated with the respective decrement in body fat mass (r = 0.49, *p* < 0.01). Similar results have been reported in other studies^50^. Only one study reported changes in endurance performance in female soldiers. Warr et al.^53^ observed a small (3%) but statistically insignificant mean improvement in endurance performance with a trivial ES (*g* 0.16, 95% CI − 0.61 to 0.94) in 12 female soldiers deployed to Afghanistan/Iraq. The improvement in females was statistically significant when compared to the respective negative change in male soldiers.

**Figure 2:**
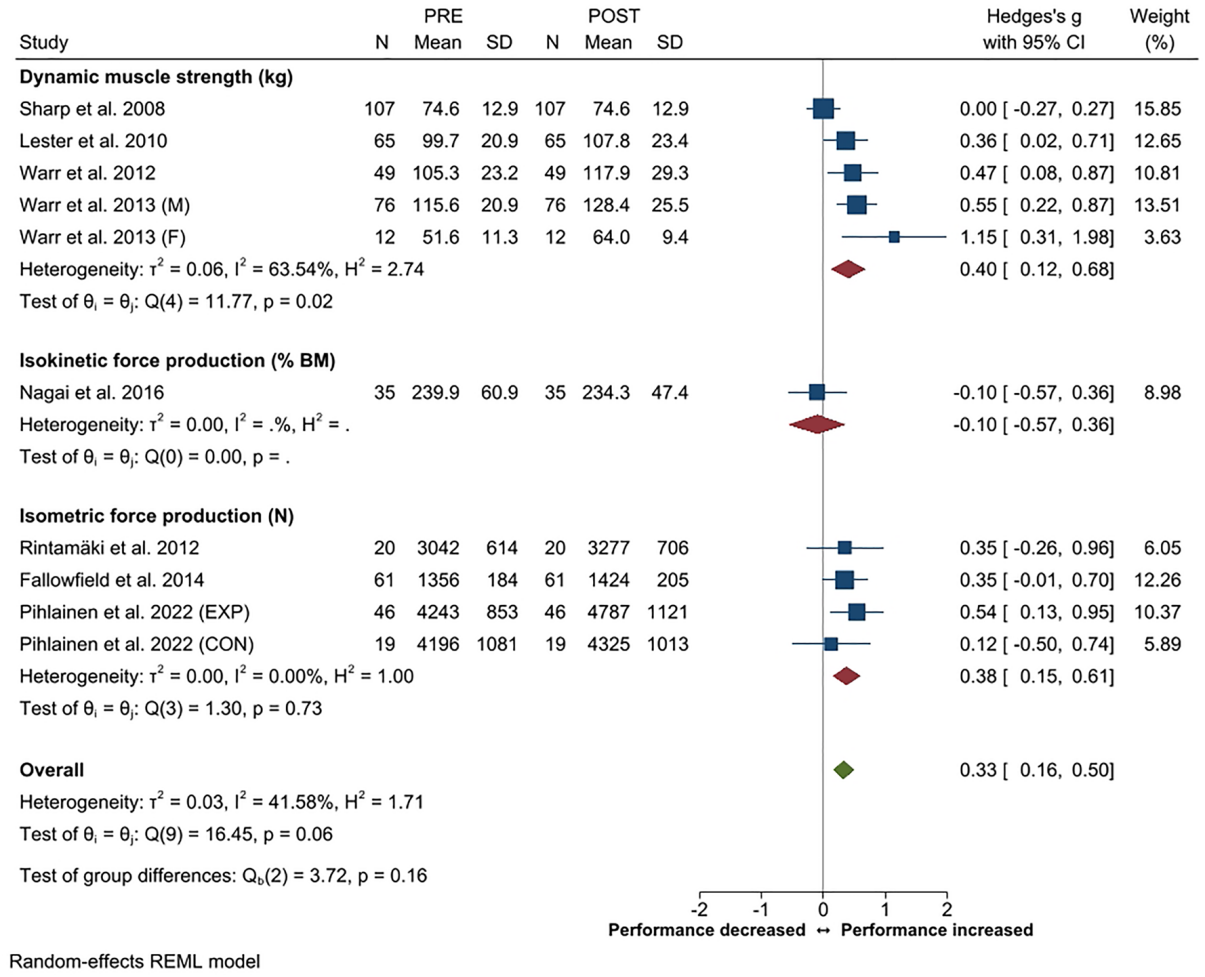
Summary of meta-analysis results for changes in maximal strength of the lower body reported in standardized means (with 95% CI) and Hedge’s g. Abbreviations; M, male; F, female; BM, body mass; EXP, experimental group; CON, control group.

**Figure 3:**
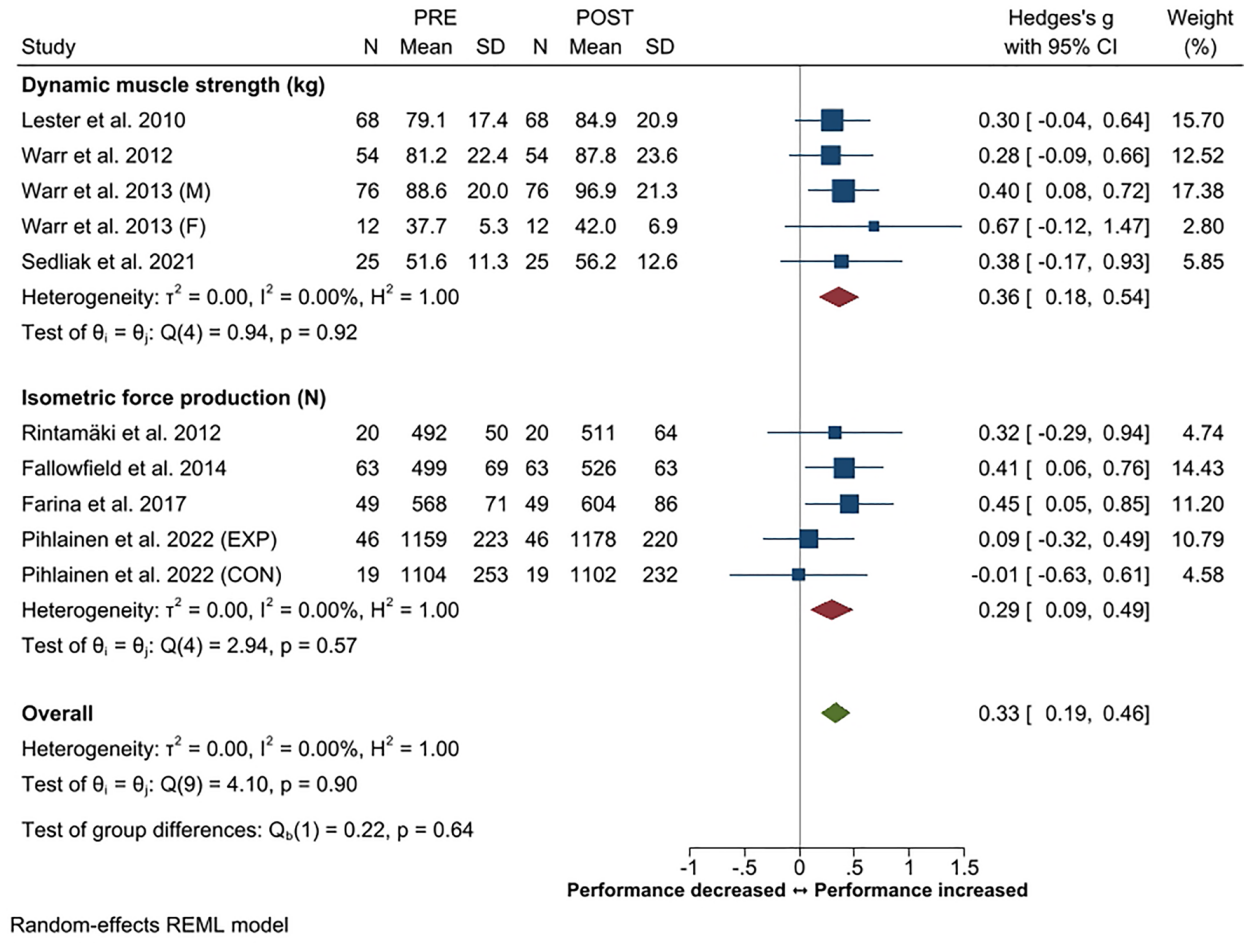
Summary of meta-analysis results for changes in maximal strength of the upper body reported in standardized means (with 95% CI) and Hedge’s g. Abbreviations; M, male; F, female; EXP, experimental group; CON, control group.

Of the available eight studies, an increase in maximal strength of the lower body was reported in five studies while none of the studies reported decreases. The overall increase in lower body strength (*g* 0.33, 95% CI 0.16–0.50) was small but statistically significant (Fig. 2) and the heterogeneity was low (42%). The sub-group analysis showed moderate heterogeneity across studies using various dynamic lower body muscle strength tests. Similarly, increase in upper body strength (Fig. 3) was reported in five out of available eight studies with small but significant overall ES for change (*g* 0.33, 95% CI 0.19–0.46). Heterogeneity across the studies assessing upper body strength was low (0%).

**Figure 4:**
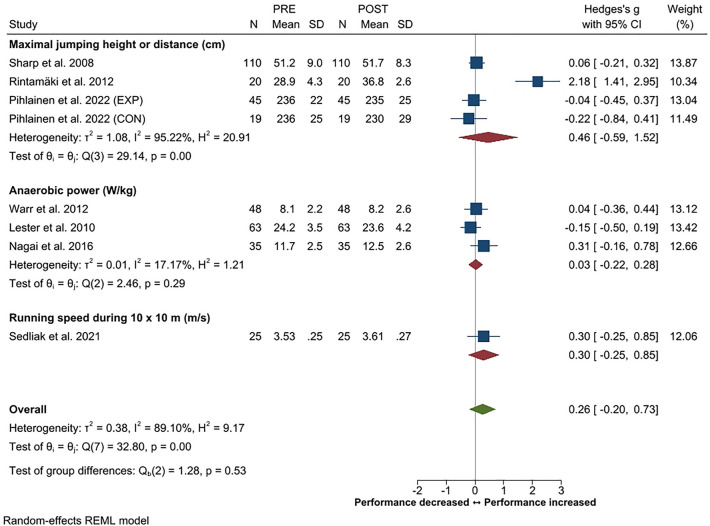
Summary of meta-analysis results for changes in lower body power reported in standardized means (with 95% CI) and Hedge’s g. Abbreviations; EXP, experimental group; CON, control group.

A measure of lower body power was reported in seven articles with a small overall *g* of 0.26 (95% CI − 0.20 to 0.73) and heterogeneity of 89%, revealing high variability especially across studies using various jump tests. With the exception of one study^55^, the changes were small to trivial (Fig. 4) and without the study of Rintamäki et al.^55^ ES would have been 0.03 (95% CI − 0.12 to 0.18). A measure of upper body power was reported only in three studies^19,21,54^ and thus, it was not included in this review.

**Figure 5:**
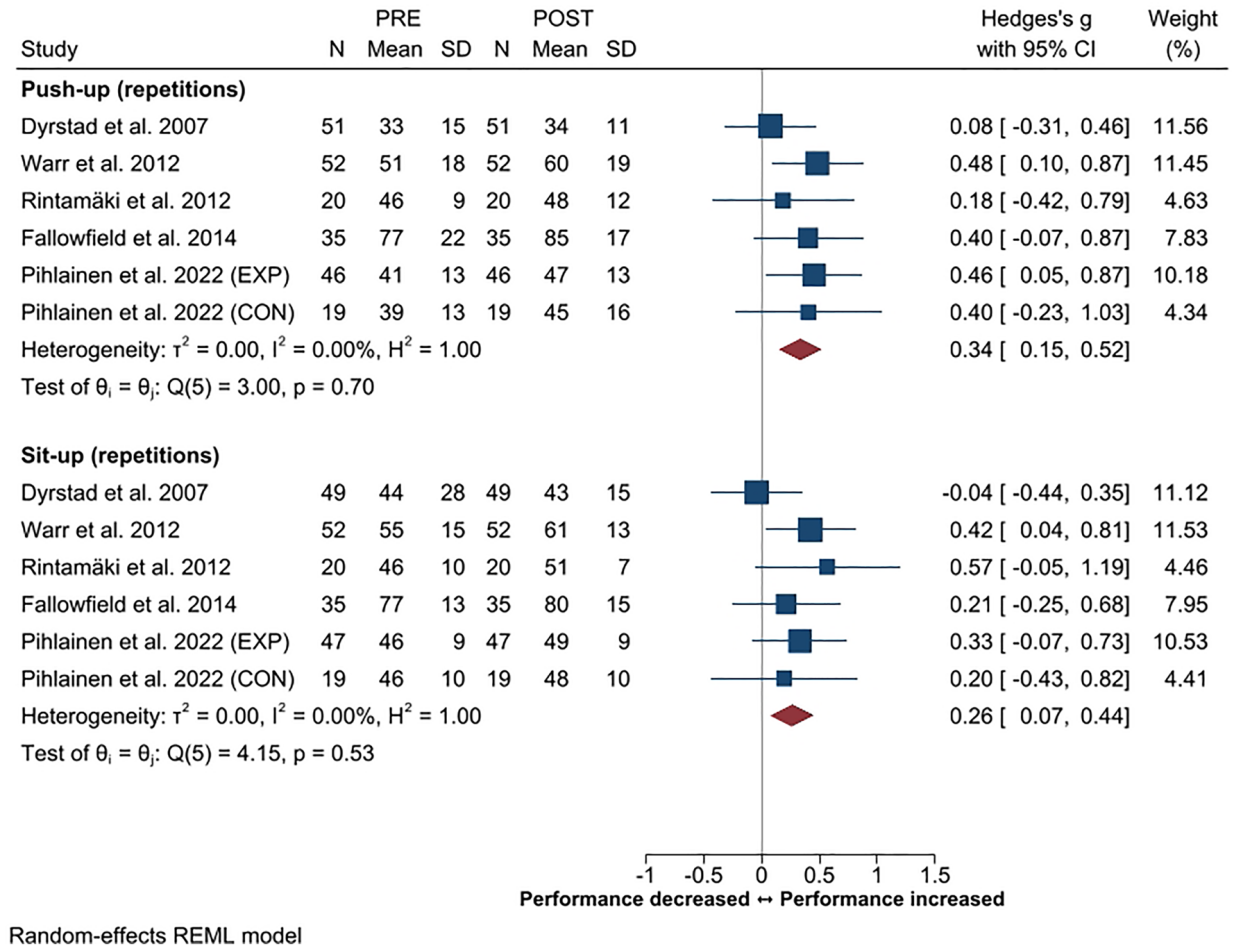
Summary of meta-analysis results for changes in muscular endurance (push-up and sit-up tests) reported in standardized mean (with 95% CI) and Hedge’s g. Test duration; Dyrstad et al. 2007, RM; Warr et al. 2012, Fallowfield et al. 2014, 2 min RM; Rintamäki et al. 2012, Pihlainen et al. 2022, 1 min RM. Abbreviations; EXP, experimental group; CON, control group.

Improvement in, at least, one muscular endurance test result (e.g., number of repetitions in pull-ups, push-ups and sit-ups) was reported in five out of six available studies^18,21,47,51,55^, while the rest reported no changes. Push-ups and sit-ups were the most commonly used tests. The overall ESs for push-up and sit-up performances were small, *g* 0.34 (95% CI 0.15–0.52) and 0.26 (95% CI 0.07–0.44), respectively (Fig. 5). The overall as well as sub-group heterogeneity was 0% in the analyses of muscular endurance.

**Figure 6:**
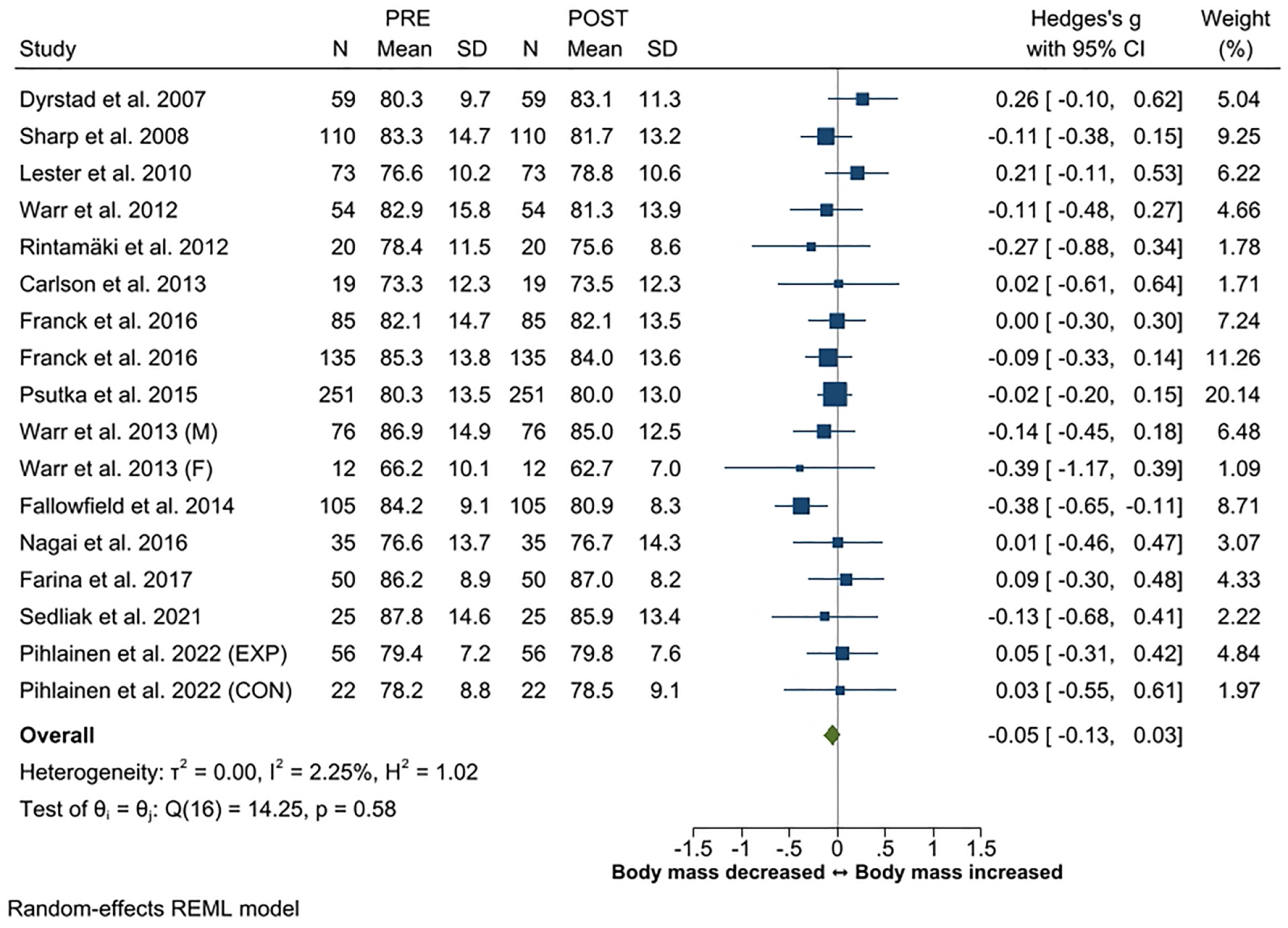
Summary of meta-analysis results for changes in body mass reported in standardized mean (with 95% CI) and Hedge’s g. Abbreviations; M, male; F, female; EXP, experimental group; CON, control group.

**Figure 7:**
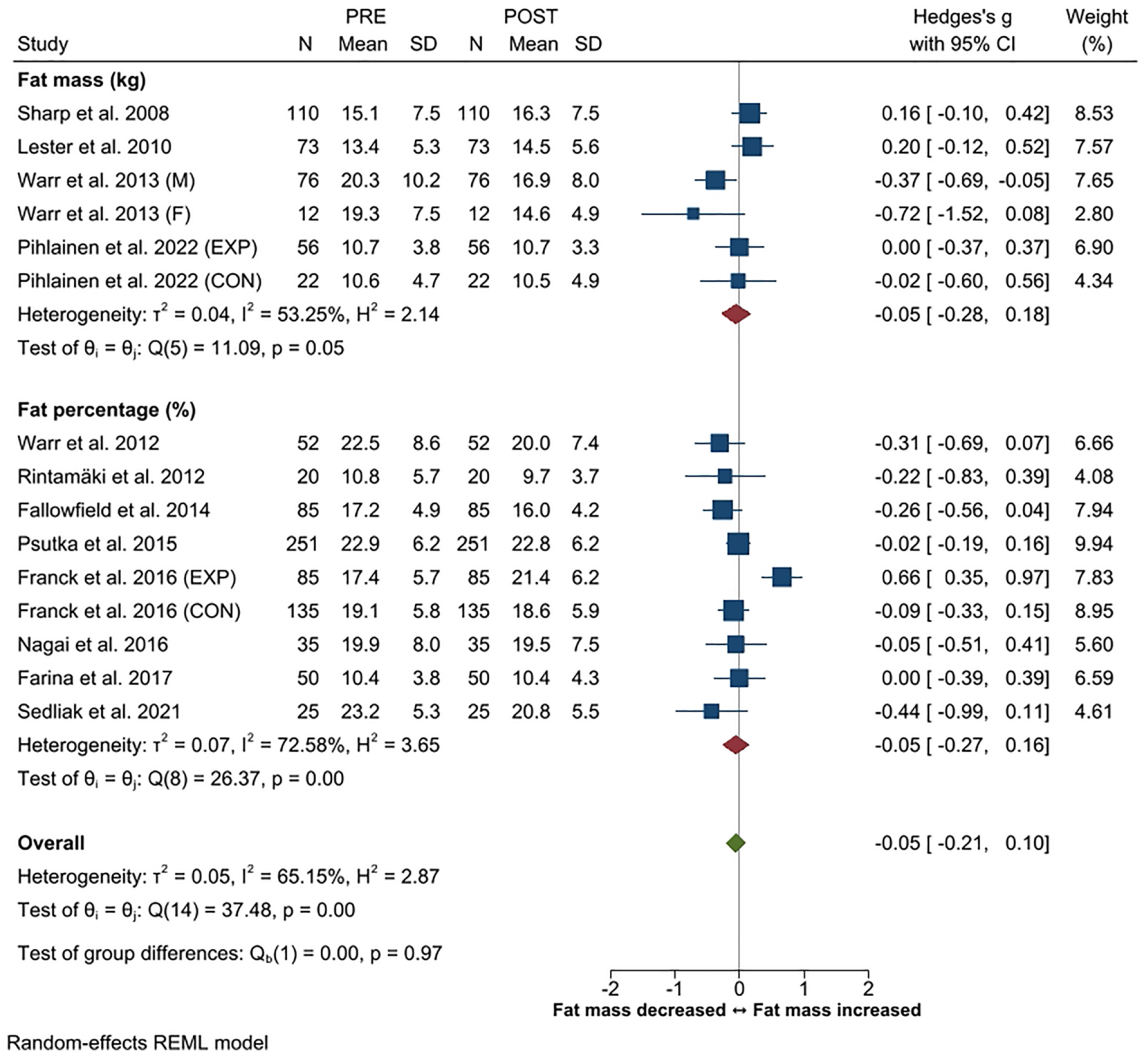
Summary of meta-analysis results for changes in fat mass reported in standardized mean (with 95% CI) and Hedge’s g. Abbreviations; M, male; F, female; EXP, experimental group; CON, control group.

**Figure 8:**
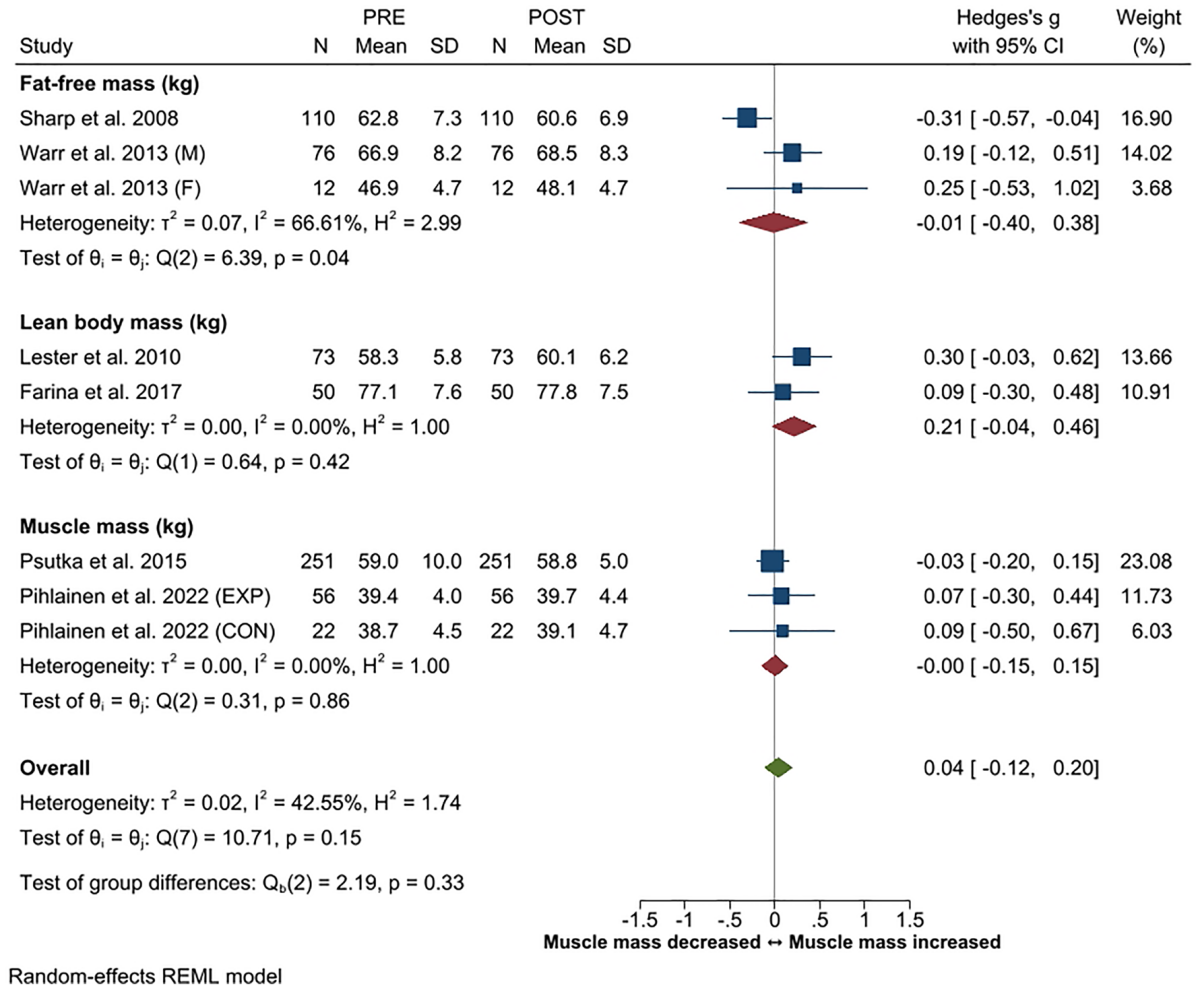
Summary of meta-analysis results for changes in muscle mass reported in standardized mean (with 95% CI) and Hedge’s g. Abbreviations; M, male; F, female; EXP, experimental group; CON, control group.

Body mass changes were reported in 15 studies, fat mass (or fat%) in 13 studies, and muscle mass (or lean mass/fat-free mass) in eight studies. The overall ES for change in body mass was trivial (*g* -0.05, 95% CI − 0.13 to 0.03). Fat mass (or fat%) increased in three studies^19,54,56^, decreased in three studies^21,51,53^, and no changes were observed in seven studies^16,20,47–49,55,57^. Overall, the ES for change in fat mass was trivial with an overall *g* of − 0.05 (95% CI − 0.21 to 0.10). Likewise, the overall ES for change in muscle mass (*g* 0.04, 95% CI − 0.12 to 0.20) was trivial. Muscle mass increased in four studies^49,53,54,57^ and in the experimental training group of one study^47^, while decreases were observed in one^19^ study. No changes in muscle mass were observed in two studies^20,48^ and in the control group of the study of Pihlainen et al.^47^. Effect sizes for body composition are presented in Figs. 6, 7 and 8.

Physical activity or training was self-reported with various methods by using training diaries or post-deployment surveys in 13 articles. Compared to pre-measurement, physical activity was maintained or decreased during the operation^49,52,56^. In addition, large inter-individual variations in physical training volume and decrements, especially in endurance training frequency and volume, were observed in the most studies that reported training statistics both preceding and during operation^19,50,54^. For example, Sharp et al.^19^ reported that prevalence of soldiers engaging endurance training, at least, three times per week reduced from 80% preceding the operation to 35% during the operation in Afghanistan. Lester et al.^54^ documented similar reductions (88% vs. 29%) from Iraq. The percentage of soldiers performing strength training, at least, three times per week did not change as markedly as endurance training. The respective pre versus during distributions for strength training were 58% versus 56%^19^ and 63% versus 44%^54^. However, one study^57^ reported increases of 60% (156 ± 106 vs. 250 ± 182 min/week) and 77% (190 ± 101 vs. 336 ± 251 min/week) in endurance and strength training volumes of 35 Special Operations Forces soldiers during 3–6 month deployment in Afghanistan and other undefined locations. Summary of physical activity and training during deployments is presented in Table 2.

Decrements in training volume were often related to negative changes in physical performance and body composition following the principle of training specificity (Table 2). Dyrstad et al.^18^ observed large individual variations in training volume and changes in aerobic capacity (r = 0.46, *p* < 0.001) during a 12-month deployment in Kosovo. One third (n = 20) of the Norwegian soldiers, whose VO_2_max decreased (− 3 ± 4%), performed physical training on average 77 ± 48 min/week (endurance 31 ± 22, strength 46 ± 35 min/week) during the operation. In contrast, the other third (n = 19) whose VO_2_max was improved (+ 4 ± 4%) during the operation, trained 169 ± 76 min per week (endurance 48 ± 46, strength 121 ± 71 min/week), e.g., more than twice as much as the group with a decreased aerobic capacity^18^. Warr et al.^53^ compared training adaptations in National Guard soldiers who engaged in training ≥ 3 times/week versus those performing less than that during deployments in Iraq and Afghanistan. In general, no changes were observed in VO_2_max during deployment, but interaction between group and time was observed between soldiers performing endurance training ≥ 3 times per week and those training less than that (∆ VO_2_max + 2% vs. − 8%, p < 0.05). Pihlainen et al.^50^ observed that deployed soldiers (n = 24) whose endurance performance decreased during a 6 month operation in Lebanon reported that their endurance training frequency was 40% lower (2.6 ± 1.6 vs. 1.4 ± 1.1 times/week) than preceding the operation. On the contrary, soldiers who improved their endurance performance during the operation, increased endurance training frequency (2.3 ± 1.4 vs. 2.4 ± 1.0 times/week) from the pre-deployment level. Also, correlation between the relative pre-during operation change in endurance training frequency and percent change in 3000 m run time was observed (r = − 0.57, *p* < 0.001).

Regarding strength training during deployment, Lester et al.^54^ reported a relationship between strength training frequency and change in 1RM bench press (r = 0.61, *p* < 0.05). Warr et al.^53^ found an interaction between group and time (*p* < 0.001) in change of 1RM bench press result between soldiers performing strength training ≥ 3 times per week (+ 15%) and those performing less than three times per week (+ 3%). Pihlainen et al.^50^ found a moderate correlation between relative pre-during operation change in strength training frequency and respective change in muscle mass (r = 0.31, *p* < 0.05). Moderate correlations between strength training frequency and changes in muscle mass have been reported also in earlier deployment studies^19,54^.

As for physical performance, also body composition changes seem to be, at least partly, related to their baseline values. For example, Sharp et al.^19^ reported significant mean decreases in body mass and fat-free mass only in soldiers in the highest initial quartiles, whereas no changes were found in the lowest quartiles. According to Farina et al.^57^, mean body mass did not change in the British Royal Marines during their 3–6 month deployment, but when participants were split into quartiles according to their baseline body mass, significant increases were observed in the lowest two groups, whereas the individuals with the highest baseline body mass tended to decrease body mass during operation.

Among the few articles reporting changes between the pre and post measurements, Fallowfield et al.^20^ observed decreases of 5% (3.9 kg) in body mass and 8% in body fat percentage in the British Royal Marines during the first half of a 6 month operational deployment in Afghanistan. The soldiers regained their body mass and fat percentage by the end of the deployment and thus, no pre-post changes were observed in these variables in the end of the study. Similarly, fat-free mass decreased by 1.9 ± 1.9 kg in the first half and increased by 2.1 ± 1.8 kg during the latter half of the operation. Pihlainen et al.^47^ also reported a modest increase in body mass during the latter half of a 6 month crisis-management operation in Lebanon in soldiers who were provided a combined strength and endurance training program (+ 0.4 kg, *p* < 0.05) as well as soldiers in the control group without training program (+ 0.7 kg, *p* < 0.05). While no pre-post changes in body mass or fat mass were observed, respective increases were reported in muscle mass (+ 0.4 kg, *p* < 0.05) of soldiers provided with a training program^47^.

## Discussion

The present systematic review and meta-analysis examined changes in physical performance and body composition during military operations with a minimum duration of three months. Additionally, the aim was to report physical activity and training along with their interactions with fitness and body composition. This meta-analysis showed that overall, endurance performance decreased while maximal strength of the lower and upper body as well as muscular endurance increased during deployments. However, the standardized mean differences were mainly trivial or small, with large variation between the studies explaining high statistical heterogeneity values. This is logical as the outcomes varied from negative to positive changes. Also, significant variation existed between the duration of deployments, measurement methods, training facilities, security situations, some to mention.

The first four available deployment studies from 2007 to 2012^18,19,51,54^ reported significant declines in endurance performance, which in general was the most consistent finding from the military operation studies of that time period regarding physical performance. Thereafter, most studies reported no change in endurance performance. However, endurance performance was the only variable in the present review showing negative overall change in terms of mean ES. Therefore, maintenance of this fitness attribute should be in the focus of military commanders during operation.

Increases in maximal strength of the lower and upper body as well as muscular endurance may reflect training habits and preferences of soldiers during deployment. The overall change with 95% CI in lower body power varied from increase to decrease. Only one study reported very large ES for increase in lower body power^55^. Excluding this result, the present meta-analysis reports no overall change in lower body power, despite the increase in maximal strength. The same study^55^ resulted in high level of heterogeneity in the meta-analysis, and it was considered as potential outlier but due to limited number of studies overall, it was not removed from the analyses. However, if it had been removed, the pooled effect size value of maximal jumping height or distance would have changed from 0.46 (− 0.59 to 1.52) to 0.00 (− 0.21 to 0.21) and, I^2^ value from 95.2 to 0.0%. Several studies have reported decrements in lower body power after strenuous military field exercise^15,58^. This may be due to neuromuscular fatigue combined with loss of muscle mass, the two factors contributing to fast force production ability. Based on findings from the reviewed deployment studies, the occupational physical load may not be as high as compared to field exercises, at least for the whole duration of the operation. Since explosive force production is important fitness component, especially, during combat missions^59^, a special focus should be paid in maintenance of this ability throughout the military operation.

The overall body composition changes were mainly small or trivial, and the results between the studies varied rather evenly from negative change to no change and positive change. A review by McCarthy et al.^60^ reported effect sizes similar to the present meta-analysis for body mass and fat%. The changes in body composition and physical performance were, at least partly, explained by individual variation in training status and history. Also, although energy balance and nutrition were not in the focus of this review, it is widely known that long-term suboptimal diet combined with insufficient physical activity may lead to negative changes in body composition, physical performance and cardiometabolic health^44^.

Expected adaptation to decrements in total training load during deployment would be attenuation of physical performance according to the principle of training specificity. This may be the major explanation for decreased endurance performance reported in many deployment studies. Especially endurance training volumes decreased in deployed soldiers^19,50,54^. In some deployments, military tasks alone were not physically demanding enough to maintain endurance performance, and the total physical activity/work load remained low^49^. It is also possible that the conditions at the military bases did not support endurance training (e.g., lack of running pathways, treadmills or ergometers, hot climate etc.). Supporting this hypothesis, some studies reported that negative change in endurance performance was associated with either lower than average endurance training frequency^19,53^ or decreased endurance training frequency from time preceding operation^50^. Thus, to avoid decrements in endurance performance, endurance-training load should not be dramatically reduced from the level preceding the operation, especially in soldiers with higher initial fitness level, who may be more susceptible to decrements in their physical performance during deployment^19,50^. In one study with a positive change in endurance performance during deployment, it was speculated that an increase in total training volume, when compared to normative training of soldiers, explained the positive adaptations^21^. Negative changes in fitness were also associated with other negative outcomes, such as higher number of medical visits^51^, increases in fat mass^18,21,50^ and decreased perception of overall health^53^.

Many of the reviewed articles, as well as other studies^17^, have reported that the overall external training load (reported training volume, frequency and/or intensity) decreased during the operation as compared to time preceding it. Many reasons may explain this phenomena, including high operational tempo, increased duties and 24 h readiness demand, lack of motivation as well as lack of training facilities or equipment. On the other hand, many studies reported that physical training occurred during operations. Thus, detraining may have been rather an individual choice than result of lacking possibilities. Indeed, one of the likely reasons is that physical training was not compulsory^18,21,47,54,55^ and maintenance of physical performance was based on the resposibility/motivation of the individual soldier. Interestingly though, many of the reviewed papers^18,21,47,54,55^ recommended more obligatory and individually tailored physical training, especially during low-tempo phases of deployment to maintain readiness and capability for the possible intensive phases and in general, until the end of the operation period. It must be acknowledged though, that intrinsic, instead of extrinsic, motivation supported maintenance of training habits during operation^18^. However, it is challenging to change intrinsic motivation towards training during deployment. One simple solution could be guided or supervised compulsory physical training, in addition to general or military specific fitness and body composition assessments, implemented throughout the deployment period. Professionally guided training sessions along with performance assessments might lead to higher motivation, more optimal adaptations, and lower injury rate. It is important to acknowledge that large proportion (23%) of non-combat injuries have been reported to occur during sport activities^61^, especially during strength training^24^. Thus, properly guided training could therefore even enhance the occupational performance of soldiers by reducing sports-related injuries.

A recommendation for physical training periodization during deployment has been presented in the literature by Haff^34^. The starting point is two weekly strength training sessions interspersed with recovery day plus aerobic/anaerobic training session for one-to-two times per week. This is in line with a review from Spiering et al.^62^ who reported that a minimum dose for maintenance of endurance performance for 15 weeks in general adult population is two weekly training sessions. Strength and muscle mass can be maintained even for longer period (up to 32 weeks) with just one weekly strength training session and one set per exercise. Thus, maintenance of physical performance at least for 15–32 weeks is possible with reduced overall training volume (duration and/or frequency) during deployment, but training intensity should be kept high. Naturally, individualization is required since large variation in training history and fitness level exist between soldiers^63^. Smith et al.^64^ reported in their review and meta-analysis that more structured high-intensity strength and endurance training, or their combination (e.g., combined training) is more effective training method to improve endurance performance, strength, power, muscular endurance in soldiers, compared to traditional military physical training typically consisting of running/walking and calisthenics with lower intensities. Moreover, high-intensity interval training (HIIT) and/or high-intensity functional training (HIFT) can be considered as a practical training method for soldiers whenever time allocated to training and access to fitness facilities are limited^65^. HIIT/HIFT may even be performed in operational environments or during operations where decrements in aerobic performance have been observed^63^. In addition, these “non-traditional” training practices may reduce musculoskeletal injury risk^64^.

Ideally, training emphasis and prescription would be based on occupational task/demands analysis. It also needs to be taken into consideration that soldiers may encounter several external stressors during deployment which may impair their ability to recover from training load and therefore, more recovery may be required for periodization of training during deployment. If these additional stressors are not considered, risks for non-functional overreaching and injury are increased^3,34,63^. One potential option is flexible non-linear periodization model, which allows soldiers to take into consideration the occupational stress and modulate training accordingly. Flexible periodization does not mean that workouts are selected by personal preferences but instead, sessions that are targeted to develop qualities that require training in a recovered state (e.g., maximal and explosive strength), are performed accordingly. If the occupational duties do not enable longer (one-hour) consecutive training sessions, they may also be performed in shorter bouts (i.e., micro-training) without the risk of attenuated training adaptations^66^.

Following limitations of reviewed studies were identified. A large variation in research methodology including differences in measurement methods, delay between post measurements and unclearly reported sex-distribution complicate the inference of results. Moderator analyses to determine which factors contribute to the variability in effect sizes may have improved the quality of the present meta-analysis. However, due to the complexity of moderating factors across the studies, this could not be performed thoroughly. Thus, conflicting findings between the studies are likely, at least partly, explained by differences in security situation, resources, possibilities, and motivation for physical training, as well as duration of the follow-up. In addition, decrements in study population was common finding of the reviewed studies. This needs to be considered when planning future military operation studies. Finally, this review did not consider the effects of nutrition in body composition or physical performance changes since studies focusing on effects of nutrition during deployment are still scarce.

## Conclusion

Each deployment is a unique challenge for soldiers to maintain their initial fitness level and body composition, which requires individually tailored training programs for optimization of physical performance and readiness throughout the operation. Overall, special attention should be paid in maintenance of aerobic endurance, which was the most likely performance variable to decrease during deployment. Regarding neuromuscular performance, lower and upper body strength and muscular endurance are less likely to decrease. Body composition changes were mainly small and varied from negative to positive changes in muscle and fat mass. Detraining seems to be problem, especially, for soldiers with high initial fitness level. To minimize declines in performance and readiness, soldiers should be encouraged to perform frequent endurance and strength training, depending on their pre-deployment training status, at least 2–4 times per week using flexible non-linear periodization. At least, a part of physical training should be supervised or preferably guided to optimize training adaptations and minimize injury risk. Finally, the number of peer-reviewed articles on changes in body composition and fitness changes and, especially in physical training during military operation is still limited. Thus, more deployment studies are warranted.

### Supplementary Information

Supplementary Table S1.

## Data Availability

This systematic review and meta-analysis has no original data to provide as they have been compiled from previously published journal articles. Most of the data have been reported within the main text and are available for copying for further analyses.

## References

[1] Henning PC, Park BS, Kim JS (2011). Physiological decrements during sustained military operational stress. Mil. Med..

[2] Sharp, M., Patton, J. & Vogel, J. A database of physically demanding tasks performed by U.S. army soldiers. Military Performance Division. U.S. Army Research Institute of Environmental Medicine, Natick MA. 1998. Technical Report T98. Available at: https://apps.dtic.mil/sti/pdfs/ADA338922.pdf. Accessed April 6, 2023.

[3] Nindl BC, Castellani JW, Warr BJ, Sharp MA, Henning PC, Spiering BA, Scofield DE (2013). Physiological Employment Standards III: Physiological challenges and consequences encountered during international military deployments. Eur. J. Appl. Physiol..

[4] Vaara JP, Groeller H, Drain J, Kyröläinen H, Pihlainen K, Ojanen T, Connaboy C, Santtila M, Agostinelli P, Nindl BC (2022). Physical training considerations for optimizing performance in essential military tasks. Eur. J. Sport Sci..

[5] Nindl BC, Alvar BA, Dudley JR, Favre MW, Martin GJ, Sharp MA, Warr BJ, Stephenson MD, Kraemer WJ (2015). Executive summary from the National strength and conditioning association's second blue ribbon panel on military physical readiness: Military physical performance testing. J. Strength Cond. Res..

[6] Friedl KE, Knapik JJ, Häkkinen K, Baumgartner N, Groeller H, Taylor NA, Duarte AF, Kyröläinen H, Jones BH, Kraemer WJ, Nindl BC (2015). Perspectives on aerobic and strength influences on military physical readiness: report of an international military physiology roundtable. J Strength Cond Res..

[7] Knapik J, Staab J, Bahrke M, Reynolds K, Vogel J, O'Connor J (1991). Soldier performance and mood states following a strenuous road march. Mil. Med..

[8] Martin K, Périard J, Rattray B, Pyne DB (2020). Physiological factors which influence cognitive performance in military personnel. Hum. Factors.

[9] O'Leary TJ, Wardle SL, Greeves JP (2020). Energy deficiency in soldiers: The risk of the athlete triad and relative energy deficiency in sport syndromes in the military. Front. Nutr..

[10] Conkright WR, O'Leary TJ, Wardle SL, Greeves JP, Beckner ME, Nindl BC (2022). Sex differences in the physical performance, physiological, and psycho-cognitive responses to military operational stress. Eur. J. Sport Sci..

[11] Sekel NM, Beckner ME, Conkright WR, LaGoy AD, Proessl F, Lovalekar M, Martin BJ, Jabloner LR, Beck AL, Eagle SR, Dretsch M, Roma PG, Ferrarelli F, Germain A, Flanagan SD, Connaboy C, Haufler AJ, Nindl BC (2023). Military tactical adaptive decision making during simulated military operational stress is influenced by personality, resilience, aerobic fitness, and neurocognitive function. Front. Psychol..

[12] Church DD, Gwin JA, Wolfe RR, Pasiakos SM, Ferrando AA (2019). Mitigation of muscle loss in stressed physiology: Military relevance. Nutrients.

[13] Conkright WR, Beckner ME, Sinnott AM, Eagle SR, Martin BJ, Lagoy AD, Proessl F, Lovalekar M, Doyle TLA, Agostinelli P, Sekel NM, Flanagan SD, Germain A, Connaboy C, Nindl BC (2021). Neuromuscular performance and hormonal responses to military operational stress in men and women. J. Strength Cond. Res..

[14] Beckner ME, Main L, Tait JL, Martin BJ, Conkright WR, Nindl BC (2022). Circulating biomarkers associated with performance and resilience during military operational stress. Eur. J. Sport Sci..

[15] Heilbronn, B., Doma, K., Sinclair, W., Connor, J., Irvine-Brown, L. & Leicht, A. Acute fatigue responses to occupational training in military personnel: A systematic review and meta-analysis. *Mil. Med.* usac144 (2022). 10.1093/milmed/usac144 PMC10187475 35639912

[16] Nagai T, Abt JP, Sell TC, Keenan KA, McGrail MA, Smalley BW, Lephart SM (2016). Effects of deployment on musculoskeletal and physiological characteristics and balance. Mil. Med..

[17] Boye MW, Cohen BS, Sharp MA, Canino MC, Foulis SA, Larcom K, Smith LUS (2017). Army physical demands study: Prevalence and frequency of performing physically demanding tasks in deployed and non-deployed settings. J. Sci. Med. Sport.

[18] Dyrstad SM, Miller BW, Hallén J (2007). Physical fitness, training volume, and self-determined motivation in soldiers during a peacekeeping mission. Mil. Med..

[19] Sharp MA, Knapik JJ, Walker LA, Burrell L, Frykman PN, Darakjy SS, Lester ME, Marin RE (2008). Physical fitness and body composition after a 9-month deployment to Afghanistan. Med. Sci. Sports Exerc..

[20] Fallowfield JL, Delves SK, Hill NE, Cobley R, Brown P, Lanham-New SA, Frost G, Brett SJ, Murphy KG, Montain SJ, Nicholson C, Stacey M, Ardley C, Shaw A, Bentley C, Wilson DR, Allsopp AJ (2014). Energy expenditure, nutritional status, body composition and physical fitness of Royal Marines during a 6-month operational deployment in Afghanistan. Br. J. Nutr..

[21] Sedliak M, Sedliak P, Vaara JP (2021). Effects of 6-month military deployment on physical fitness, body composition, and selected health-related biomarkers. J. Strength Cond. Res..

[22] Swank A, Sharp C, Haff GG, Triplett T (2016). Adaptations to aerobic endurance training programs. Essentials of Strength Training and Conditioning.

[23] Häkkinen K, Alén M, Komi PV (1985). Changes in isometric force- and relaxation-time, electromyographic and muscle fibre characteristics of human skeletal muscle during strength training and detraining. Acta Physiol. Scand..

[24] Roy TC, Knapik JJ, Ritland BM, Murphy N, Sharp MA (2012). Risk factors for musculoskeletal injuries for soldiers deployed to Afghanistan. Aviat. Space Environ. Med..

[25] Drain J, Billing D, Neesham-Smith D, Aisbett B (2016). Predicting physiological capacity of human load carriage—A review. Appl. Ergon..

[26] Beckner ME, Conkright WR, Eagle SR, Martin BJ, Sinnott AM, LaGoy AD, Proessl F, Lovalekar M, Jabloner LR, Roma PG, Basner M, Ferrarelli F, Germain A, Flanagan SD, Connaboy C, Nindl BC (2021). Impact of simulated military operational stress on executive function relative to trait resilience, aerobic fitness, and neuroendocrine biomarkers. Physiol. Behav..

[27] Hauschild VD, DeGroot DW, Hall SM, Grier TL, Deaver KD, Hauret KG, Jones BH (2017). Fitness tests and occupational tasks of military interest: A systematic review of correlations. Occup. Environ. Med..

[28] Crawford K, Fleishman K, Abt JP, Sell TC, Lovalekar M, Nagai T, Deluzio J, Rowe RS, McGrail MA, Lephart SM (2011). Less body fat improves physical and physiological performance in army soldiers. Mil. Med..

[29] Lyons J, Allsopp A, Bilzon J (2005). Influences of body composition upon the relative metabolic and cardiovascular demands of load-carriage. Occup. Med. (Lond.).

[30] Pihlainen K, Santtila M, Häkkinen K, Kyröläinen H (2018). Associations of physical fitness and body composition characteristics with simulated military task performance. J. Strength Cond. Res..

[31] Szivak TK, Kraemer WJ (2015). Physiological readiness and resilience: Pillars of military preparedness. J. Strength Cond. Res..

[32] Szivak TK, Lee EC, Saenz C, Flanagan SD, Focht BC, Volek JS, Maresh CM, Kraemer WJ (2018). Adrenal stress and physical performance during military survival training. Aerosp. Med. Hum. Perform..

[33] Billing DC, Drain JR (2017). International Congress on Soldiers' Physical Performance 2017: Research priorities across the service members operational lifecycle. J. Sci. Med. Sport.

[34] Haff G, Alvar B, Sell K, Deuster P (2017). Periodization for tactical populations. NCSA´s Essentials of Tactical Strength Training and Conditioning.

[35] Page MJ, McKenzie JE, Bossuyt PM, Boutron I, Hoffmann TC, Mulrow CD, Shamseer L, Tetzlaff JM, Akl EA, Brennan SE, Chou R, Glanville J, Grimshaw JM, Hróbjartsson A, Lalu MM, Li T, Loder EW, Mayo-Wilson E, McDonald S, McGuinness LA, Stewart LA, Thomas J, Tricco AC, Welch VA, Whiting P, Moher D (2021). The PRISMA 2020 statement: An updated guideline for reporting systematic reviews. BMJ.

[36] Methley AM, Campbell S, Chew-Graham C, McNally R, Cheraghi-Sohi S (2014). PICO, PICOS and SPIDER: A comparison study of specificity and sensitivity in three search tools for qualitative systematic reviews. BMC Health Serv. Res..

[37] Lakens D (2013). Calculating and reporting effect sizes to facilitate cumulative science: A practical primer for t-tests and ANOVAs. Front. Psychol..

[38] Nakagawa, S. & Cuthill, I. C. Effect size, confidence interval and statistical significance: A practical guide for biologists. *Biol. Rev. Camb. Philos. Soc.* **82**(4):591–605 (2007). doi: 10.1111/j.1469-185X.2007.00027.x. Erratum in: *Biol. Rev. Camb. Philos. Soc.* **84**(3):515 (2009). 10.1111/j.1469-185X.2007.00027.x 17944619

[39] Higgins JP, Thompson SG, Deeks JJ, Altman DG (2003). Measuring inconsistency in meta-analyses. BMJ.

[40] Jackson D, Turner R (2017). Power analysis for random-effects meta-analysis. Res. Synth. Methods.

[41] Hill NE, Fallowfield JL, Delves SK, Ardley C, Stacey M, Ghatei M, Bloom SR, Frost G, Brett SJ, Wilson DR, Murphy KG (2015). Changes in gut hormones and leptin in military personnel during operational deployment in Afghanistan. Obesity (Silver Spring).

[42] Farina EK, Taylor JC, Means GE, Murphy NE, Pasiakos SM, Lieberman HR, McClung JP (2017). Effects of deployment on diet quality and nutritional status markers of elite U.S. Army special operations forces soldiers. Nutr. J..

[43] Fallowfield JL, Delves SK, Hill NE, Lanham-New SA, Shaw AM, Brown PEH, Bentley C, Wilson DR, Allsopp AJ (2019). Serum 25-hydroxyvitamin D fluctuations in military personnel during 6-month summer operational deployments in Afghanistan. Br. J. Nutr..

[44] Nykänen T, Pihlainen K, Santtila M, Vasankari T, Fogelholm M, Kyröläinen H (2019). Diet macronutrient composition, physical activity, and body composition in soldiers during 6 months deployment. Mil. Med..

[45] Nykänen T, Pihlainen K, Kyröläinen H, Fogelholm M (2020). Associations of nutrition and body composition with cardiovascular disease risk factors in soldiers during a 6-month deployment. Int. J. Occup. Med. Environ. Health.

[46] Pihlainen K, Pesola AJ, Helén J, Häkkinen K, Finni T, Ojanen T, Vaara JP, Santtila M, Raitanen J, Kyröläinen H (2020). Training-induced acute neuromuscular responses to military specific test during a six-month military operation. Int. J. Environ. Res. Public Health.

[47] Pihlainen K, Kyröläinen H, Santtila M, Ojanen T, Raitanen J, Häkkinen K (2022). Effects of combined strength and endurance training on body composition, physical fitness, and serum hormones during a 6-month crisis management operation. J. Strength Cond. Res..

[48] Psutka J, Pavlík V, Fajfrová J, Urban M, Halajčuk T (2015). Monitoring of anthropometric changes in the armed forces of the Czech Republic personnel during the deployment in Afghanistan. Mil. Med. Sci. Let..

[49] Pihlainen K, Santtila M, Vasankari T, Häkkinen K, Kyröläinen H (2018). Evaluation of occupational physical load during 6-month international crisis management operation. Int. J. Occup. Med. Environ. Health.

[50] Pihlainen K, Häkkinen K, Santtila M, Raitanen J, Kyröläinen H (2020). Differences in training adaptations of endurance performance during combined strength and endurance training in a 6-month crisis management operation. Int. J. Environ. Res. Public Health.

[51] Warr BJ, Heumann KJ, Dodd DJ, Swan PD, Alvar BA (2012). Injuries, changes in fitness, and medical demands in deployed National Guard soldiers. Mil. Med..

[52] Carlson, A. R., Smith, M. A. & McCarthy, M. S. Diet, physical activity, and bone density in soldiers before and after deployment. *US Army Med. Dep. J.* 25–30 (2013). 23584905

[53] Warr BJ, Scofield DE, Spiering BA, Alvar BA (2013). Influence of training frequency on fitness levels and perceived health status in deployed National Guard soldiers. J. Strength Cond. Res..

[54] Lester ME, Knapik JJ, Catrambone D, Antczak A, Sharp MA, Burrell L, Darakjy S (2010). Effect of a 13-month deployment to Iraq on physical fitness and body composition. Mil. Med..

[55] Rintamäki H, Kyröläinen H, Santtila M, Mäntysaari M, Simonen R, Torpo H, Mäkinen T, Rissanen S, Lindholm H (2012). From the subarctic to the tropics: Effects of 4-month deployment on soldiers' heat stress, heat strain, and physical performance. J. Strength Cond. Res..

[56] Frank LL, McCarthy MS (2016). Telehealth coaching: Impact on dietary and physical activity contributions to bone health during a military deployment. Mil. Med..

[57] Farina EK, Taylor JC, Means GE, Williams KW, Murphy NE, Margolis LM, Pasiakos SM, Lieberman HR, McClung JP (2017). Effects of combat deployment on anthropometrics and physiological status of U.S. army special operations forces soldiers. Mil. Med..

[58] Nindl BC, Leone CD, Tharion WJ, Johnson RF, Castellani JW, Patton JF, Montain SJ (2002). Physical performance responses during 72 h of military operational stress. Med. Sci. Sports Exerc..

[59] Mala, J., Szivak, T. K., Flanagan, S. D., Comstock, B. A., Laferrier, J. Z., Maresh, C. M., & Kraemer, W. J. The role of strength and power during performance of high intensity military tasks under heavy load carriage. *US Army Med. Dep. J.* 3–11 (2015). 26101902

[60] McCarthy MS, Elshaw EB, Szekely BM, Pflugeisen B (2017). Health promotion research in active duty army soldiers: The road to a fit and ready force. Nurs. Outlook.

[61] Sanders JW, Putnam SD, Frankart C, Frenck RW, Monteville MR, Riddle MS, Rockabrand DM, Sharp TW, Tribble DR (2005). Impact of illness and non-combat injury during Operations Iraqi Freedom and Enduring Freedom (Afghanistan). Am. J. Trop. Med. Hyg..

[62] Spiering BA, Mujika I, Sharp MA, Foulis SA (2021). Maintaining physical performance: The minimal dose of exercise needed to preserve endurance and strength over time. J. Strength Cond. Res..

[63] Kyröläinen H, Pihlainen K, Vaara JP, Ojanen T, Santtila M (2018). Optimising training adaptations and performance in military environment. J. Sci. Med. Sport.

[64] Smith C, Doma K, Heilbronn B, Leicht A (2022). Effect of exercise training programs on physical fitness domains in military personnel: A systematic review and meta-analysis. Mil. Med..

[65] Helén J, Kyröläinen H, Ojanen T, Pihlainen K, Santtila M, Heikkinen R, Vaara JP (2023). High-intensity functional training induces superior training adaptations compared with traditional military physical training. J. Strength Cond. Res..

[66] Kilen A, Bay J, Bejder J, Breenfeldt Andersen A, Bonne T, Larsen P, Carlsen A, Egelund J, Nybo L, Vidiendal Olsen N, Aachmann-Andersen NJ, Løvind Andersen J, Nordsborg NB (2021). Distribution of concurrent training sessions does not impact endurance adaptation. J. Sci. Med. Sport.

